# The Impact of SARS-CoV-2 Viral Load on the Mortality of Hospitalized Patients: A Retrospective Analysis

**DOI:** 10.7759/cureus.16540

**Published:** 2021-07-21

**Authors:** António Machado, Pedro Salvador, Pedro Oliveira, Tiago Teixeira, Cristóvão Figueiredo, Sofia Nunes, Luís Silva, Leonor Silva, Tiago Costa, Luís Malheiro

**Affiliations:** 1 Department of Medicine, Faculdade de Medicina da Universidade do Porto, Porto, PRT; 2 Internal Medicine Department, Centro Hospitalar de Vila Nova de Gaia/Espinho, Vila Nova de Gaia, PRT; 3 Infectious Diseases Department, Centro Hospitalar de Vila Nova de Gaia/Espinho, Vila Nova de Gaia, PRT; 4 Infectious Diseases Department, Centro Hospitalar de Vila Nova de Gaia/Espinho, Vila Nova De Gaia, PRT; 5 Pathology and Laboratory Medicine Department, Centro Hospitalar de Vila Nova de Gaia/Espinho, Vila Nova de Gaia, PRT; 6 Internal Medicine Department, Centro Hospitalar de Vila Nova de Gaia/Espinho, Vila Nova De Gaia, PRT

**Keywords:** sars-cov-2, covid-19, viral load, mortality, viral shedding

## Abstract

Introduction

Coronavirus disease 2019 (COVID-19) has emerged worldwide since December 2019. The standard method for diagnosis is via nucleic acid amplification testing, usually with a reverse-transcription polymerase chain reaction (RT-PCR). Hospitalized infected individuals may require ventilation and may have higher mortality rates. We aim to evaluate the clinical impact of nasopharyngeal viral load on these outcomes.

Materials and methods

We conducted a retrospective cohort study of patients hospitalized with COVID-19 from 17 March 2020 to 1 June 2020 at a tertiary care hospital. Severe acute respiratory syndrome coronavirus 2 (SARS-CoV-2) viral load was assessed using cycle threshold (Ct) values from an RT-PCR assay applied to nasopharyngeal swab samples. We compared the clinical characteristics of survivors vs. non-survivors and assessed whether the viral load was independently associated with in-hospital 30-day mortality.

Results

We evaluated 197 patients. Thirty-day mortality was verified in 71 (36%) subjects. In the adjusted effects model, only the *E*-gene Ct value [odd ratio (OR) .873; confidence interval (CI) 95% .769-.992; p .037], age, the number of days of symptoms before admission, lactate dehydrogenase (LDH), and the oxygen saturation (SatO_2_)-to-fraction of inspired oxygen (FiO_2_) ratio remained significantly associated with 30-day mortality. There was no identified association between the viral loads and disease severity, the need for ventilation, or length of stay.

Discussion

Our results are, in part, concordant with previous papers. One possible limitation to our study is the fact that possibly included disproportionately more patients with poorer outcomes since hospitalization was required. Therefore, further research is required.

Conclusion

SARS-CoV-2 viral load on admission may be an independent predictor of 30-day mortality among hospitalized patients with COVID-19. Providing this information to clinicians could potentially be used to guide risk stratification.

## Introduction

In December 2019, several cases of pneumonia of unknown etiology were described in the city of Wuhan, Hubei Province, China [1]. By the 7th of January 2020, a novel coronavirus, originally abbreviated as 2019-nCoV by World Health Organization (WHO), was identified in the throat swab samples of 15 of the 59 cases of pneumonia. Soon after, the first novel coronavirus genome sequence was released to the scientific community [2]. The pathogen was later renamed as severe acute respiratory syndrome coronavirus 2 (SARS-CoV-2) by the Coronavirus Study Group and the disease was named coronavirus disease 2019 (COVID-19) by WHO. By the end of January 2020, WHO declared the novel coronavirus outbreak as a public health emergency of international concern. At that time, 7,736 confirmed and 12,167 suspected cases had been reported in China and 82 confirmed cases had been detected in 18 other countries. As of 10th of March 2021, 117,332,262 cases of COVID-19 had been reported worldwide, including 2,605,356 deaths [3].

By virtue of being highly contagious, SARS-CoV-2 has quickly disseminated within all continents. It has been confirmed that COVID-19 presents a human-to-human transmission via droplets, and possibly by aerosols and direct contact [4]. The disease presentation may range from asymptomatic to severe pneumonia and death, and overall mortality ranges from 0.25% to as high as 3.0% [5].

The standard method for COVID-19 diagnosis is via nucleic acid amplification testing (NAAT), usually with a real-time reverse-transcription polymerase chain reaction (RT-PCR) assay for the detection of SARS-CoV-2 RNA in respiratory samples. RT-PCR cycle threshold (Ct) represents the number of replication cycles required for sufficient gene amplification to produce a fluorescent signal that crosses a predefined threshold and correlates inversely with quantitative viral load. Therefore, the Ct value can provide an indirect method for estimating the number of copies of viral RNA in a sample swab. It has been suggested that the viral load of SARS-CoV-2 may be an important factor in determining the disease severity and progression [6]. However, further research is needed to identify clinical determinants of the SARS-CoV-2 viral load and the clinical importance of the Ct value.

The main purpose of this study was to evaluate if there is an association between the SARS-CoV-2 Ct value (as a proxy for viral load) and outcomes such as 30-day mortality and disease severity in hospitalized patients due to COVID-19.

## Materials and methods

Patients and study design

This study was performed in accordance with the Strengthening the Reporting of Observational Studies in Epidemiology (STROBE) reporting guidelines. We performed a retrospective cross-sectional study in a cohort of subjects with COVID-19, admitted to Centro Hospitalar Vila Nova de Gaia/Espinho (CHVNG/E) between March 17, 2020 and June 1, 2020. Inclusion criteria were age ≥ 18 years old, need for hospitalization, and confirmed COVID-19 by a positive NAAT for SARS-CoV-2 in a nasopharyngeal (NP) sample within the 48-hours prior to admission. The exclusion criteria were the unavailability of a Ct value for SARS-CoV-2 NAAT or if NAAT testing was performed in another laboratory and not repeated within 24-hours of admission. Patients who tested positive for SARS-CoV-2 due to nosocomial outbreaks were included in the analysis, as were asymptomatic patients admitted due to non-clinical reasons such as the need for isolation measures, the lack of social support, and clinical conditions unrelated to COVID-19. The study was approved by the local ethics committee (reference 124/2020).

Data collection

The subjects were identified using the CHVNG/E COVID-19 Registry database. Data were retrospectively retrieved from the electronic medical records by the investigators. The retrieved data included demographics, comorbidities, selected outpatient medications, presenting symptoms on admission to the hospital, laboratory parameters, and in-hospital mortality. Demographic features included sex, age, and source of admission. Comorbidities were the presence of arterial hypertension, obesity, malignancies, chronic kidney disease (CKD), diabetes mellitus (DM), chronic cardiovascular diseases (CVD), chronic lung disease (CLD), and any immunosuppression. Selected outpatient medications on admission included angiotensin receptor blockers or angiotensin-converting enzyme inhibitors (ARB/ACEi) and statins.

The clinical presentation of COVID-19 was based on compatible symptoms upon admission, including cough, fever, dyspnoea, chest pain, diarrhoea, and haemoptysis. Admitted patients were categorized according to disease severity as asymptomatic, mild/moderate (absence of dyspnoea or presence of dyspnoea without hypoxia), severe (dyspnoea and hypoxia requiring supplemental oxygen), and critical (respiratory failure, shock, or multiple organ dysfunction) diseases.

The main outcome was determined by reviewing discharge summaries. We assumed that patients were survivors if they were discharged alive and there was no readmittance in the 30 days after discharge with no further description of death. Secondary outcomes were disease severity, length of stay, and the need for ventilation (invasive or non-invasive mechanical ventilation).

Laboratorial analysis

For all inpatients, haematology and biochemical results were retrieved from the electronic records of the included subjects. Only results obtained within 24 hours of admission were considered. Haematology data included serum haemoglobin (Hb), red cell distribution width (RDW), total leukocyte, lymphocyte, and platelet absolute counts. A neutrophil-to-lymphocyte index was obtained by dividing the absolute neutrophil count by the lymphocyte count. Biochemical parameters included serum creatinine, lactate dehydrogenase (LDH), alanine transaminase (TGP), serum albumin, total serum bilirubin, troponin I, C-reactive protein (CRP), Interleukin-6 (IL-6), procalcitonin, ferritin, fibrinogen, arterial lactate, international normalized ratio (INR), N-terminal prohormone of brain natriuretic peptide (NT-pro BNP), and D-dimers.

Viral load assessment

SARS-CoV-2 infection was diagnosed using the Cobas® SARS-CoV-2 test on the Roche Cobas® 6800 system (Roche Diagnostics, Rotkreuz, Switzerland). This test is a single-well dual-target assay, which includes both specific detection of SARS-CoV-2 and pan-Sarbecovirus detection for the Sarbecovirus subgenus family that includes SARS-CoV-2 [7]. Selective amplification of target nucleic acid from the sample is achieved by using target-specific primers for ORF1 a/b non-structural region that is specific to SARS-CoV-2, and primers for the conserved region in the structural protein envelope *E*-gene, which is common to all Sarbecovirus. Results were considered positive or reactive if both the *ORF1 a/b* and/or *E*-gene were detected, and as presumptive, if only the *E*-gene was amplified in the presence of a compatible clinical syndrome. The Ct value for each target was retrospectively collected by an experienced investigator.

Statistics

Baseline characteristics are presented as proportions (%) if categorical, as mean (standard deviation) if continuous with normal distribution, or as median [interquartile range (Q1-Q3)] if continuous with non-normal distribution. For the main objective of the study, the Ct values of SARS-CoV-2 RT-PCR results were compared between survivors and non-survivors. Other patient characteristics were also compared between the groups. Univariate analysis of differences between groups was performed with χ^2^ proportion comparison test for proportions (%), 2-sample, 2-sided t-test for means, and 2-sided Mann-Whitney U test for medians. Differences in means between >2 categories were calculated with Tukey's honest significance test. Multivariable analysis using logistic regression was performed for the risk factors found to be significantly associated with mortality in the univariate analysis, and taking into consideration possible interactions between variables. Odds ratio (OR) with 95% confidence interval (CI) were used to report the association between mortality and exposure to the risk factors. The pattern of independent variables missing data was analysed to minimize the bias caused by missing values. Only variables with more than 80% available results were considered in the multivariate model. All statistical analysis was performed using Statistical Package for Social Sciences (SPSS) version 26 (IBM Corp., Armonk, New York). A p-value < .05 was considered statistically significant.

## Results

During the study period, 243 patients were admitted due to COVID-19. Forty-six patients were excluded due to the unavailability of the Ct value for the NP swab. The final study population included 197 patients whose characteristics can be found in Table 1. The source of admission was mostly from outside the hospital (home or nursing home) with only 6 (3.1%) of the included patients turning positive during hospital admission for another clinical reason. A total of 148 (75.2%) patients had severe of critical disease. Out of the remaining 49 (24.9%) patients with asymptomatic or mild/moderate disease, the reason for admission was the lack of social support or home isolation measures in 35 (17.7%) patients, an acute surgical/orthopedic condition in 8 (4.1%), and an acute medical condition unrelated to COVID-19 in 6 (3.1%) patients. Our sample population had a median age of 80 years old (range 30-100 years old), with 104 (52.8%) male patients. The source of admission was home in 124 (62.9%) patients, a long-term healthcare facility in 67 (34%) patients, or another hospital department in 6 (3.1%) patients. About 173 (87.8%) patients had at least one relevant pre-existing comorbidity; with arterial hypertension, diabetes mellitus, and obesity being the most common. The most reported symptoms upon admission were fever, cough, and dyspnoea, which were present in 144 (73.1%), 124 (62.9%), and 114 (57.9%) patients, respectively.

**Table 1:** Univariate analysis of factors influencing 30-day mortality Results are presented as ^α^incidence rates (%) if categorical, ^Ω^mean (standard deviation), or ^β^median (Q1-Q3). Legend: AST - aspartate transaminase; CRP - C-reactive protein; Ct – cycle threshold; IL-6 - interleukin-6; INR - international normalized ratio; LDH - lactate dehydrogenase; NT-proBNP - N-terminal fragment in the prohormone brain natriuretic peptide; RDW - red cell distribution width; SatO_2 _- oxygen saturation; PaO_2_ - arterial oxygen partial pressure; FiO_2_ - fraction of inspired oxygen

	Total (n=197)	Survivors (n=126)	Non-survivors (n=71)	p-value
Demographics, comorbidities, and clinical parameters				
Male^α^	104 (52.8)	64 (50.8)	40 (56.3)	.454
Female^α^	93 (47.2)	62 (49.2)	31 (43.7)	
Age^β^	80 (66-86)	75 (63-84)	85 (80-88)	< .001
Any comorbidity^α^	173 (87.8)	106 (84.1)	67 (94.4)	.035
Arterial hypertension^α^	140 (71.1)	84 (66.7)	56 (78.9)	.007
Obesity^α^	39 (19.8)	30 (23.8)	9 (12.7)	.060
Malignancy^α^	19 (9.6)	7 (5.6)	12 (16.9)	.010
Chronic Kidney Disease^α^	30 (15.2)	19 (15.1)	11 (15.5)	.938
Diabetes Mellitus^α^	60 (30.5)	38 (30.2)	22 (31.0)	.904
Cardiovascular Disease^α^	35 (17.8)	24 (19.0)	11 (15.5)	.078
Chronic Lung Disease^α^	15 (7.6)	7 (5.6)	8 (11.3)	.147
Immunosuppression^α^	6 (3.0)	5 (4.0)	1 (1.4)	.315
Days of symptoms before admission^α^	3 (1; 7)	4.5 (2; 8.3)	3 (1; 4)	.001
Cough^α^	114 (57.9)	73 (57.9)	41 (57.7)	.979
Fever^α^	144 (73.1)	94 (74.6)	50 (70.4)	.525
Dyspnoea^α^	124 (62.9)	68 (54.0)	56 (78.9)	.001
Chest Pain^α^	20 (10.2)	16 (12.7)	4 (5.6)	.115
Diarrhoea^α^	25 (12.7)	19 (15.1)	6 (8.5)	.180
Haemoptysis^α^	4 (2.0)	0 (0.0)	4 (5.6)	.016
SatO_2_/FiO_2_ ratio^β^	428 (354; 447)	438 (419; 447)	395 (267; 432)	< .001
PaO_2_/FiO_2_ ratio^β^	274 (223; 318)	283 (241; 319)	242 (186; 314)	.016
Disease severity^α^				< .001
Asymptomatic	22 (11.2)	20 (15.9)	2 (2.8)	
Mild/Moderate	27 (13.7)	25 (19.8)	2 (2.8)	
Severe	125 (63.5)	63 (50.0)	62 (87.3)	
Critical	23 (11.7)	18 (14.3)	5 (7.0)	
Laboratorial results				
*Orf1* Ct^Ω^	24.9 (5.4)	25.95 (0.46)	22.98 (0.65)	< .001
*E* Ct^Ω^	26.0 (6.2)	27.15 (0.53)	23.88 (0.72)	< .001
Haemoglobin (g/gL)^Ω^	12.5 (2.1)	12.74 (0.19)	12.11 (0.25)	.050
RDW (%)^β^	13.8 (12.9; 14.7)	13.4 (12.7; 14.4)	14.2 (13.2; 15.8)	.001
Leukocytes (x10^3^/µL)^β^	7.7 (5.15; 10.84)	6.57 (4.70; 9.06)	9.38 (6.46; 15.57)	< .001
Lymphocytes (x10^3^/µL)^β^	1.1 (0.73; 1.43)	1.08 (0.83; 1.44)	0.94 (0.63; 1.37)	.161
Neutrophil-to-Lymphocyte index^β^	5.6 (3.3; 9.0)	4.95 (2.92; 7.54)	6.94 (4.57; 14.47)	< .001
Platelets (x10^3^/µL)^Ω^	220 (118))	216 (8.66)	227 (18.38)	.530
Serum creatinine (mg/dL)^β^	1.03 (0.79; 1.54)	0.95 (0.76;1.33)	1.21 (0.85;1.74)	.021
LDH (mg/dL)^Ω^	344 (10.42)	325 (10.67)	384 (22.72)	.009
AST (U/mL)^β^	24.0 (16.0; 41.0)	27.0(17.0; 50.0)	19.0 (15.0; 33.5)	.007
Total bilirrubin (mg/dL)^β^	0.42 (0.3; 0.6)	0.43 (0.36;0.64)	0.36 (0.27;0.49)	.004
Serum albumin (g/L)^Ω^	3.4 (0.53)	3.60 (0.09)	3.11 (0.09)	< .001
Troponin I (ng/L)^β^	27.0 (14.0; 60.0)	18.0 (9.5; 40.5)	50.0 (38.0; 94.0)	.001
INR^β^	1.12 (1.07; 1.25)	1.11 (1.07; 1.21)	1.19(1.08; 1.34)	.017
D-dimer (µg/mL)^β^	1.55 (0.98; 2.89)	1.38 (0.80; 2.82)	3.05 (1.27; 4.18)	.006
CRP (mg/dL)^β^	9.01 (3.38; 15.19)	10.26 (5.89; 14.92)	15.49 (9.22; 19.70)	.014
IL-6 (pg/mL)^β^	59.6 (31.1; 152.0)	62.15 (26.7; 152.0)	98.8 (40.0; 161.0)	.064
Ferritin (ng/mL)^β^	810 (413; 1468)	764.5 (408.0; 1329.0)	819.0 (515.0; 1292.0)	.521
NT-ProBNP (pg/mL)^β^	899 (174; 2813)	428 (144; 1294)	3718(2499; 6915)	< .001
Fibrinogen^Ω^	575 (183)	563 (191)	604 (166)	.341
Procalcitonin^β^	0.24 (0.12; 0.66)	0.17(0.08;0.52)	0.43(0.20;1.02)	.007
Arterial lactate^β^	0.8 (0.7; 1.3)	0.8(0.7;1.2)	1.1(0.7;1.6)	.014

Factors influencing viral load (*E* and *Orf* genes) and time-to-negativity (TTN)

The *E* gene was amplified in 197 (100%) patients, while the *Orf1 *gene was amplified in 191 (96.9%) patients. There was a strong and significant correlation between the Ct of the *E* and the *Orf1* genes (R -.990, p <.001). The *E* and *Orf1* gene Ct varied significantly with age (p .002 and p <.001, respectively), the time between symptoms onset and testing (p .001, for both). The *Orf1* Ct also varied significantly in the presence of hypertension (p .034). The evaluation of other factors influencing the *E* and *Orf1 *Ct can be found in Table 2. There were no other identified differences of the *E* and *Orf1* Ct when comparing sex, symptoms, and other comorbidities.

**Table 2:** Univariate analysis of clinical factors influencing SARS-CoV-2 viral load Results are presented as *median (Interquartilic range) or ^¥^Pearson's R. In categorical categories, results are presented as present vs non-present. Legend: ARB/ACEi - angiotensin receptor antagonists/angiotensin-converting enzyme inhibitors; CKD – chronic kidney disease; Ct – cycle threshold; CVD – cardiovascular disease; DM – diabetes mellitus; TTN – time to negativity.

	*E* Ct (viral load)	p-value	*Orf1* Ct (viral load)	p
Age^ ¥^	-.233	.002	-.252	< .001
Male^ *^	25.7 (8.77)	0.343	25.1 (8.11)	0.298
Female^ *^	25.7 (10.77)		25.4 (9.97)	
Days of symptoms until test^ ¥^	.248	.001	.266	.001
Arterial hypertension^ *^	25.4 (8.65) vs 26.4 (12.8)	.063	14.7 (7.92) vs 25.9 (11.1)	.034
Obesity ^*^	26.2 (10.9) vs 25.7 (9.5)	.770	25.4 (9.59) vs 25.1 (8.82)	.772
Cancer^ *^	23.0 (6.5) vs 26.0 (9.8)	.138	22.6 (6.1) vs 25.5 (9.0)	.106
CKD^ *^	27.6 (11.1) vs 25.6 (9.7)	.752	26.9 (9.7) vs 25.1 (8.9)	.455
DM^ *^	23.1 (10.3) vs 26.2 (9.6)	.076	22.6 (9.7) vs 25.6 (8.7)	.117
CVD^ *^	26.2 (8.1) vs 25.5 (10.2)	.415	25.9 (7.5) vs 24.8 (9.4)	.600
Resp. disease^ *^	25.7 (9.1) vs 25.7 (9.7)	.932	25.1 (8.3) vs 25.2 (9.1)	.857
ARB/ACEi^ *^	25.5 (8.5) vs 25.9 (10.7)	.325	25.0 (7.8) vs 25.4 (9.8)	.368
Statin^ *^	25.9 (9.8) vs 25.7 (9.7)	.456	25.4 (8.9) vs 24.9 (8.9)	.430
Disease severity^ *^		.362		.351
Asymptomatic	23.6 (12.8)		22.9 (12.3)	
Mild-Moderate	28.8 (7.9)		27.9 (7.5)	
Severe	25.7 (9.5)		25.1 (8.7)	
Critical	25.8 (11.9)		25.2 (10.8)	
Length of stay^ ¥^	.056	.434	.025	.734
TTN^ ¥^	-.259	.018	-.209	.066
Need of ventilation^*^	25.8 (5.9) vs 25.6 (6.04)	.962	24.9 (5.42) vs 24.8 (5.43)	.918

**Figure 1:**
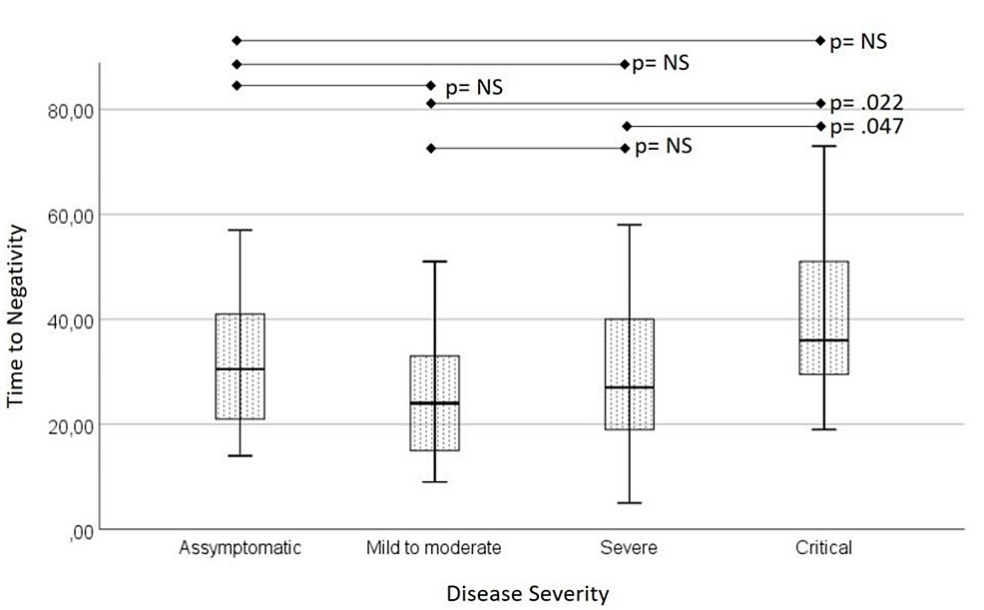
Evaluation of time-to-negativity between different disease severities.

In 82 (41.7%) patients, sequential NP swabs were performed on a weekly basis until a negative result was achieved. In the 115 patients in whom it was not possible to obtain a negative swab, the reasons were, death in 63 (31.9%) patients and loss of follow-up in 52 (26.4%) patients. The mean time-to-negativity (TTN) was 31 (SD 1.6) days, ranging from 5 to 73 days. The TTN correlated weakly with the initial *ORF1* gene Ct (R -.209, p .066) but significantly with the *E* gene Ct (R -.259, p .018) on admission. We did not find any association between Ct values and demographics, comorbidities, or clinical parameters. When considering disease severity, patients with critical disease had significantly longer TTN than patients with mild-moderate disease (Figure 1).

Evaluation of outcomes

The main outcome (30-day mortality) was verified in 71 (36%) subjects. Table 1 summarizes the clinical characteristics of survivors and non-survivors. Compared with survivors, non-survivors were significantly older [median age, 85 years (interquartile range or IQR 80-88) vs 75 years (IQR 63-84); p <.001] and were more likely to have an underlying comorbidity, specifically arterial hypertension [56 (78.9%) vs 84 (66.7%); p .007] and malignancies [12 (16.9%) vs 7 (5.6%); p .01]. Non-survivors were also more likely to present with dyspnoea [56 (78.9%) vs 68 (54.0%); p .001], haemoptysis [4(5.6%) vs 0; p .016] and shorter duration of symptoms before admission [3 (IQR 1 to 4 days) vs 4.5 (2 to 8.3 days); p .001]. Additionally, statistically significant differences between survivors and non-survivors were found in biochemical results and clinical parameters (Table 1). Non-survivors presented on admission with lower Ct values (higher viral loads) for both the *E *and *ORF1* genes (p <.001 for both), lower haemoglobin values (p .050), higher RDW (%) (p .001), higher leukocyte counts (p<.001), higher neutrophil-to-lymphocyte indexes (p <.001), higher serum creatinine (p .021), higher lactate dehydrogenase (p .009), higher serum troponin I (p .001), higher INR (p .017), higher D-dimers (p .006), higher C-reactive protein (p .014), higher NT-proBNP (p <.001), higher procalcitonin (p .007), and higher arterial lactate (p .014). Non-survivors were also more likely to present with lower oxygen saturation (SatO_2_) or arterial oxygen partial pressure (PaO_2_)-to-fraction of inspired oxygen (FiO_2_) ratio (p <.001 and p .016, respectively). However, most patients did not present with respiratory failure. None of the patients were treated with remdesivir or received corticosteroids directed to the treatment of COVID-19.

In the adjusted effects model, only age, the number of days of symptoms before admission, the *E*-gene Ct value, LDH, and the SatO_2_-to-FiO_2_ ratio remained significantly associated with 30-day mortality (Table 3). Although a significant difference was found in the univariate analysis, we excluded troponin I, procalcitonin, NT-proBNP, and D-dimers from this analysis as they were not systematically collected and less than 80% of the subjects had an available result. The SatO_2_-to-FiO_2_ ratio was chosen over the PaO_2_-to-FiO_2_ due to its wider availability. The *E*-gene Ct values were included over *ORF1*, as it was invariably quantified in every subject. There was no identified association between the *E* and *Orf1* gene viral loads and the secondary outcomes: disease severity, the need for ventilation, or length of stay.

**Table 3:** Multivariate analysis of factors influencing 30-day mortality OR - odds ratio; CI - confidence interval; CRP - C-reactive protein; Ct – cycle threshold; INR - international normalized ratio; LDH - lactate dehydrogenase; RDW - red cell distribution width.

	OR	95% CI	p-value
Age	1.119	1.023-1.225	.014
Days of symptoms before admission	.759	.609-.945	.014
*E* gene Ct	.873	.769-.992	.037
Haemoglobin	1.115	.715-1.740	.631
Leukocyte count	1.111	.893-1.383	.343
Neutrophil-to-lymphocyte index	1.012	.906-1.132	.828
RDW (%)	1.192	.838-1.696	.328
Creatinine	1.245	.644-2.405	.515
INR	1.242	.853-1.809	.259
CRP	1.071	.979-1.172	.133
Arterial hypertension	.616	.092-4.149	.619
Cancer	1.661	.235-11.740	.611
Lactate	.937	.246-3.564	.924
LDH	1.009	1.002-1.016	.008
SatO_2_-to-FiO_2_ ratio	.986	.975-.998	.018

## Discussion

In this retrospective cross-sectional study, we reported that patients with higher SARS-CoV-2 NP viral load on admission were more likely to die during their hospitalization. Even after adjusting for other confounding factors, SARS-CoV-2 Ct values were predictive of mortality in hospitalized patients with COVID-19, suggesting that NP viral loads may be relevant in the early risk stratification and identification of patients with higher odds for mortality.

Our findings are supported by previous reports that have shown SARS-CoV-2 Ct values in NP swabs to be predictive of disease severity and worse outcomes [8-11]. SARS-CoV-2 Ct in NP swabs is an appealing method as it is easily accessible and used in most algorithms for COVID-19 diagnosis on hospital admission. Some studies, however, failed to demonstrate an association between SARS-CoV-2 Ct and outcomes and raised questions about whether NP viral loads may be universally used to assess disease prognosis [12-14]. Reasons include a lack of standardization between RT-PCR for SARS-CoV-2 as available commercial tests may amplify different targets, yielding different results. Sampling variability may also introduce errors as different sampling procedures may retrieve different Ct as the amount of virus in the swab is directly dependent on the sampling approach (i.e., type and quality of the specimen procedure) [15]. Sampling normalization using a marker for the cell mass or the mucosal surface sampled should be integrated into commercial diagnostic kits to make the different assays comparable. This would allow sequential tests in the same patient to be comparable and further studies to identify cut-off values that could aid in the identification of at-risk patients [15]. Although our findings seem promising, it should be noted that SARS-CoV-2 Ct values should be considered a snapshot that has to be contemporized in the natural history of the disease. Viral load is known to decrease after symptom onset, and the longer the time between the first day of symptoms and NAAT testing, the lower the expected viral loads and sensitivity of the essay [16]. As seen in our results, the SARS-CoV-2 Ct values are significantly associated with the timing in which the test was performed, so that both factors were independent predictors of mortality, suggesting that they should be taken into consideration as a combination, when predicting outcomes.

Our results also highlighted age as an independent predictor of mortality. These results agree with findings from previous studies demonstrating that elderly people have higher odds for severe disease and mortality [17]. Age-dependent impairment of T and B cell function and excess production of type 2 cytokines are believed to lead to a deficiency in control of viral replication and more prolonged proinflammatory responses, potentially leading to poorer outcomes in the elderly [18]. Furthermore, aging is associated with a well-known innate and adaptive immune systems weakening associated with a chronic proinflammatory state [19]. Additionally, elderly patients have a decreased cardiorespiratory reserve and are more likely to have comorbidities including atherosclerosis, which may render them more susceptible to complications and possibly higher viral loads [20]. These factors may contribute to a “viral-friendly” environment, where viral replication is enhanced.

Some other studies have also found SARS-CoV-2 viral loads in NP samples to be predictive of overall disease severity, degree of hypoxemia and the risk of mechanical ventilation in COVID-19 [6, 8, 21]. An explanation for this association could be that the up-regulation of angiotensin-converting enzyme 2 receptor (ACE2R) after infection may lead to the production of cytokines such as IL-1, IL-10, and IL-6, and the regulation of B cell activation is triggered to respond. Higher expression of ACE2R could increase the penetration of the virus into the host cell and enhance viral replication, further promoting a more severe pattern of disease with acute respiratory distress syndrome (ARDS) and systemic inflammation [22].

However, in our results, we could not find a clear association between initial SARS-CoV-2 Ct values and clinical criteria of disease severity or the need for mechanical ventilation. Our results are supported by other studies that have also failed to find such association [23-25]. Some studies even found higher SARS-CoV-2 NP viral loads in less symptomatic patients when compared to hospitalized patients [13, 24]. Our results may be partially explained by the fact that the scale used to measure disease severity may not truly reflect the complexity of COVID-19. In our study, viral load failed to correlate with the need for ventilation, and the difference between classifying the patient with critical disease is directly dependent on the decision of admittance to the Intensive Care Unit (ICU). Half of the study population had ages > 80 years old and lower odds for admission to the ICU due to the high number of comorbidities, therapeutic limitation and bed shortage due to the pandemic. Therefore, the consideration of a patient as “critical” may have been associated with a selection bias that could have had influenced our results. Furthermore, in severe and critical COVID-19, ARDS is characteristic of a later phase of the disease in which viral loads are expected to be lower and inflammatory response is increased. As in the beginning of the pandemic, it was mandatory for patients to stay at home if mildly symptomatic, they would present to the emergency department and be tested later in the disease course, when symptoms progress and viral load decreases.

In 82 patients, sequential NP swabs were performed on a weekly basis until a negative result was achieved. The mean time-to-negativity (TTN) was 31±1.6 days which is also supported by previous studies [14, 26]. We did not find any association between the TTN and demographic, comorbidities, or clinical parameters, except when considering disease severity. In fact, patients who presented with critical disease had significantly longer TTN than asymptomatic or patients with mild-moderate disease. In a systematic review on SARS-CoV-2 viral load dynamics, 20 studies evaluated the duration of viral RNA shedding based on disease severity [27]. Thirteen of these studies reported a longer duration of viral shedding in patients with severe illness when compared to those with non-severe illness. Nonetheless, there is evidence that positive RT-PCR tests for SARS-CoV-2 may not be correlated to the infection state if the sample is taken later in the disease course. Wölfel et al. demonstrated that SARS-CoV-2 could not be isolated in cell culture after 8 days from the symptom onset, despite ongoing high viral load in RT-PCR results [16]. Similarly, Bullard et al. concluded that specimens with high Ct values or taken >8 days after the symptom onset were not able to infect cells [28]. Therefore, even though patients with more severe disease may have more prolonged viral shedding, it may not correlate with infectivity nor ongoing viral replication.

Our results add to a body of knowledge supporting a possible association between initial NP viral load and individual prognosis in hospitalized COVID-19 patients, although other factors such as the number of days since symptoms onset, age and pneumonia severity are also independent predictors of mortality. We were able to extensively collect a wide range of patient data in a considerable number of subjects that allowed us to control multiple comorbidities. Additionally, about 25% of the included population were asymptomatic or mildly ill patients, which makes our results representative of a wide range of clinical presentations in COVID-19.

However, there are some limitations to our study. All included subjects were hospitalized, and even though we included asymptomatic and mildly ill patients, it is possible that the differences in SARS-CoV-2 viral load between survivors and non-survivors would be less striking if we had included outpatients with COVID-19. Furthermore, due to the retrospective nature of this study, data was collected from the electronic medical records and may have presented misclassified patient characteristics and outcomes. Additionally, laboratory results were not available for all patients, including arterial blood gas analysis and, therefore, some variables may have had their role underestimated in predicting 30-day mortality. Including larger cohorts of patients with COVID-19 infection from different locations would have also helped to further define the clinical characteristics and risk factors of the disease. Our study was performed before the approval of remdesivir and dexamethasone for the treatment of COVID-19, which decreases the risk of bias in our cohort as these treatments may have an impact on COVID-19 progression and prognosis [29]. Lastly, although commercially available NAAT are sensitive methods for diagnosing COVID-19, they still provide only indirect quantifications of viral load. As SARS-CoV-2 evolves, multiple mutations may occur at the position of primers or probes of the currently used target genes and affect the test sensitivity and predictive value. The interpretation of a single Ct value should be performed cautiously as it may be affected by sample collection procedure, and the adopted assay (and analysed targets). Therefore, our results may not be generalizable for other available tests [30].

## Conclusions

We demonstrated that SARS-CoV-2 NP viral load on admission, as inferred by Ct values, is independently associated with higher odds for 30-day mortality among symptomatic and asymptomatic hospitalized patients with COVID-19. These findings suggest that the initial Ct value may be a helpful predictor for the identification of patients at higher risk of severe outcomes. However, the interpretation of a single Ct value should always be performed cautiously as it may be affected by the timing of the disease course and sample collection procedure and we cannot generalize our results to other commercially available essays that amplify other gene targets. Further studies should aim to confirm our results and identify reliable Ct cut-offs to aid clinical practice.
